# Contrast-enhanced spectral mammography in neoadjuvant chemotherapy monitoring: a comparison with breast magnetic resonance imaging

**DOI:** 10.1186/s13058-017-0899-1

**Published:** 2017-09-11

**Authors:** Valentina Iotti, Sara Ravaioli, Rita Vacondio, Chiara Coriani, Sabrina Caffarri, Roberto Sghedoni, Andrea Nitrosi, Moira Ragazzi, Elisa Gasparini, Cristina Masini, Giancarlo Bisagni, Giuseppe Falco, Guglielmo Ferrari, Luca Braglia, Alberto Del Prato, Ivana Malavolti, Vladimiro Ginocchi, Pierpaolo Pattacini

**Affiliations:** 1 Radiology Unit, Department of Diagnostic Imaging and Laboratory Medicine, Arcispedale Santa Maria Nuova - IRCCS, Viale Umberto I, No. 50, 42123 Reggio Emilia, Italy; 2 Radiology Unit, Department of Diagnostic Imaging and Laboratory Medicine, AUSL Reggio Emilia, via Amendola 2, Reggio Emilia, 42122 Italy; 3 Medical Physics Unit, Department of Oncology and Advanced Technologies, Arcispedale Santa Maria Nuova - IRCCS, Viale Umberto I, No. 50, 42123 Reggio Emilia, Italy; 4 Pathology Unit, Department of Oncology and Advanced Technologies, Arcispedale Santa Maria Nuova - IRCCS, Viale Umberto I, No. 50, 42123 Reggio Emilia, Italy; 5 Oncology Unit, Hospital C. Magati, AUSL Reggio Emilia, Via Martiri della Libertà, No. 8, Scandiano (RE), 42019 Italy; 6 Oncology Unit, Department of Oncology and Advanced Technologies, Arcispedale Santa Maria Nuova - IRCCS, Viale Umberto I, No. 50, 42123 Reggio Emilia, Italy; 7 Breast Surgery Unit, Department of Surgery, Arcispedale Santa Maria Nuova - IRCCS, Viale Umberto I, No. 50, 42123 Reggio Emilia, Italy; 8 Scientific Directorate, Arcispedale Santa Maria Nuova - IRCCS, Viale Umberto I, No. 50, 42123 Reggio Emilia, Italy

**Keywords:** Breast Cancer, Neoadjuvant chemotherapy, Treatment monitoring, CESM (contrast enhanced spectral mammography), Dual-energy, Contrast media, MRI (magnetic resonance imaging)

## Abstract

**Background:**

Neoadjuvant-chemotherapy (NAC) is considered the standard treatment for locally advanced breast carcinomas. Accurate assessment of disease response is fundamental to increase the chances of successful breast-conserving surgery and to avoid local recurrence. The purpose of this study was to compare contrast-enhanced spectral mammography (CESM) and contrast-enhanced-MRI (MRI) in the evaluation of tumor response to NAC.

**Methods:**

This prospective study was approved by the institutional review board and written informed consent was obtained. Fifty-four consenting women with breast cancer and indication of NAC were consecutively enrolled between October 2012 and December 2014. Patients underwent both CESM and MRI before, during and after NAC. MRI was performed first, followed by CESM within 3 days. Response to therapy was evaluated for each patient, comparing the size of the residual lesion measured on CESM and MRI performed after NAC to the pathological response on surgical specimens (gold standard), independently of and blinded to the results of the other test. The agreement between measurements was evaluated using Lin’s coefficient. The agreement between measurements using CESM and MRI was tested at each step of the study, before, during and after NAC. And last of all, the variation in the largest dimension of the tumor on CESM and MRI was assessed according to the parameters set in RECIST 1.1 criteria, focusing on pathological complete response (pCR).

**Results:**

A total of 46 patients (85%) completed the study. CESM predicted pCR better than MRI (Lin’s coefficient 0.81 and 0.59, respectively). Both methods tend to underestimate the real extent of residual tumor (mean 4.1mm in CESM, 7.5mm in MRI). The agreement between measurements using CESM and MRI was 0.96, 0.94 and 0.76 before, during and after NAC respectively. The distinction between responders and non-responders with CESM and MRI was identical for 45/46 patients. In the assessment of CR, sensitivity and specificity were 100% and 84%, respectively, for CESM, and 87% and 60% for MRI.

**Conclusion:**

CESM and MRI lesion size measurements were highly correlated. CESM seems at least as reliable as MRI in assessing the response to NAC, and may be an alternative if MRI is contraindicated or its availability is limited.

## Background

Preoperative neoadjuvant chemotherapy (NAC) is considered the standard treatment for locally advanced breast carcinomas to observe chemosensitivity, reduce micrometastatic diseases and for down-staging of primary tumors [1, 2].

Response to NAC is currently assessed by combining clinical examination and conventional imaging techniques such as mammography and ultrasound (US). These methods, however, often fail to predict the final pathologic response [3]. Breast contrast-enhanced magnetic resonance imaging (MRI) has proven to be superior to mammography and US in the assessment of tumor extent and presence of additional foci (i.e. multifocality and/or multicentricity), and highly sensitive in identifying residual disease following NAC [4–6]. Contrast-enhanced spectral mammography (CESM), a promising dual-energy breast imaging technique acquired after intravenous iodine-based contrast agent, allows the evaluation of vascularized lesions in the breast, which has, up to now, been a prerogative mainly of breast MRI [7]. Compared with mammography and US, CESM improves the sensitivity for breast cancer detection without decreasing specificity [8–10]. CESM digital detector has higher spatial resolution than MRI (i.e. typically pixel sizes 100 μm and 1 mm, respectively), revealing details that are approximately 10 times better [11].

CESM is currently used for the detection of primary breast cancer, for the assessment of the extent of disease, as a problem-solving tool and as a replacement for MRI where the latter is contraindicated [12]. In terms of patient preferences and tolerance, significantly higher overall preference towards CESM has been demonstrated, due to faster procedure time, greater comfort and significantly lower rate of anxiety [13].

MRI and CESM assess treatment response by evaluating tumor size reduction and type of vascularization. In the assessment of the size of the tumor no relevant discrepancies between CESM and MRI have been observed [11, 14, 15]. However MRI involves high costs and limited availability also in industrialized countries [16]. Thus there is the rationale for substituting MRI with CESM in NAC monitoring if the performance of CESM is similar to that of MRI.

To the best of our knowledge, there are no data in the literature reporting CESM versus MRI for NAC monitoring. Therefore, the purpose of our prospective study was to compare CESM and breast MRI in the assessment of tumor response, using postoperative histologic assessment as the gold standard.

## Methods

This prospective study was performed in accordance with the Declaration of Helsinki and approved by the local ethics committee. All eligible patients were willing to undergo all study procedures and provided written informed consent prior to enrolment.

### Patients

From October 2012 to December 2014, 54 consenting women (mean age 54 years, range 33–72) were consecutively enrolled in this prospective study. Inclusion criteria were age 18 years or older and histologically proven breast cancer with indications for NAC. Exclusion criteria were: known BRCA-mutation (to ensure the highest radioprotection); pregnancy; contraindications to MRI or contraindications to CESM, including a history of an anaphylactoid or anaphylactic reaction to any contrast medium or impaired renal function with chronic kidney disease stage 3 or higher (creatinine clearance < 60 ml/min).

The diagnosis of breast carcinoma was based on a percutaneous tru-cut biopsy to determine the histological and molecular characteristics of the lesion detected at the initial multimodality assessment based on physical examination, digital mammography and ultrasound. The therapeutic pathway of each patient was discussed and planned by the Breast Unit Multidisciplinary Group. The NAC protocol included the administration of anthracycline for the first 3 months and then taxanes for the next 3 months; trastuzumab was added in cases of human epidermal growth factor receptor 2 (HER2)^+^ carcinoma. Before NAC, each patient was tattooed to ensure correct surgical positioning of the region to be excised [17].

### Study workflow

**Fig. 1:**
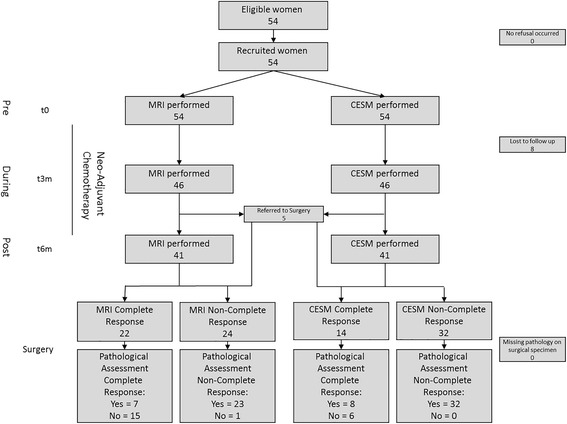
Flow chart of the study. MRI magnetic resonance imaging, CESM contrast-enhanced spectral mammography

The study workflow is shown in Fig. 1.

CESM and MRI were performed thrice: time (t)0, before the beginning of NAC (pre-NAC); t3m, after about 3 months (during-NAC) and t6m, after the end of treatment (post-NAC), just before surgery. In premenopausal women, MRI and CESM were performed between the 5^th^ and 12^th^ day after the end of menstrual flux. MRI was performed first, and then CESM was performed within 3 days. According to the first MRI performed before the beginning of NAC, if the neoplasm involved both breasts, then CESM was performed bilaterally, otherwise it was performed only on the affected breast.

### CESM protocol

CESM is based on a dual-energy system developed by GE Healthcare (Chalfont St-Giles, UK): after contrast administration, a set of low-energy and high-energy images is acquired in quick succession while the breast remains compressed, obtaining a *low*-*dose image*, comparable to a standard digital mammogram, and a post-processing *recombined image*, which enhances the distribution of the iodine contrast medium. The three digital mammography units (GE Senographe Essential) used in this study were equipped with molybdenum (Mo) and rhodium (Rh) anode tracks and filters, which can be combined to perform standard mammography. Additional copper (Cu) filters were installed to allow the combinations of Mo/Cu or Rh/Cu and shape the X-ray spectra for CESM dual-energy exposures. The examinations were performed using the CESM automatic exposure option, set according to the thickness of the compressed breast and its density. The average glandular dose (AGD) delivered by the CESM examinations was evaluated.

The CESM protocol was designed according to Dromain et al. [9]. The intravenous contrast agent was administered by a nurse under the supervision of a radiologist, using a hand-held battery-powered injector (Optistat, Covidien). The contrast agent was ioversolo 350 mg/ml (Optiray, Covidien) and the administered dose was 1.5 ml/kg of body weight [7–16]. In a monolateral CESM, the radiographer compressed the breast for the medio-lateral oblique projection (MLO) 2 minutes after administration of contrast agent and then decompressed the breast, and after a further 2 minutes again compressed the breast for the cranio-caudal projection (CC).

### MRI protocol

MRI examinations were performed with three different MRI scanners (Philips-Achieva 1.5 T, Siemens-Avanto 1.5 T and GE-Signa 1.5 T), all equipped with phased-array coils (seven coils on the Philips-Achieva; eight coils on the Siemens-Avanto and GE-Signa). The same MRI protocol was applied in the prone position and with no breast compression. The sequences acquired were: T2-weighted with or without fat suppression; 3D dynamic perfusion T1-weighted with or without fat suppression, with a first acquisition before contrast agent administration (mask) and seven acquisitions after the gadolinium contrast agent was administered, and temporal resolution between 50 and 70 s; diffusion weighted sequence where b values of 0 and 600 mm/s^2^ were set. The images were then processed using commercial software (Cadstream, Merge Healthcare) [18–20]. A detailed assessment of the enhancement kinetics was evaluated by the radiologist especially during diagnosis, but its description is not an objective of the study as the comparison is based on measurements of the size of the enhanced lesions.

### Endpoint of the study and image analysis

A total of seven independent radiologists, all experts in breast imaging (from 5 to 18 years’ experience), took part in this study to evaluate and report CESM and MRI examinations. In the clinical workflow, the radiologist evaluated either CESM or MRI of the single patient, blinded to the MRI or CESM performed at the same step of the study, but not blinded to all previous breast examinations.

The endpoint of the study was the comparison of post-NAC measurements on CESM and MRI, with the histopathological response identified in surgical specimens considered as the gold standard. Correlation between the measurements of the lesion using the two diagnostic tools was then evaluated for each step of the study (pre NAC, during NAC and post NAC). And last of all, the response to therapy was assessed for each patient according to the parameters set in the response evaluation criteria in solid tumors (RECIST) 1.1 criteria, considering the sum of the largest dimension of malignancies at baseline and its variation at subsequent measurements [3, 21]. Response was classified as follows: complete response (CR, disappearance of all lesions); partial response (PR, ≥ 30% dimensional reduction), stable disease (SD, < 30% dimensional reduction/< 20% dimensional increase) and progressive disease (PD, ≥ 20% dimensional increase).

In CESM measurements were obtained considering the maximum dimension on recombined images, based on contrast uptake. Since the low-energy image of CESM allows the visualization and characterization of microcalcifications, this information, integrating the extension of the enhanced area [16], contributed to defining the size of the tumor bed, especially in the pre-NAC evaluation.

In MRI, the maximum diameter of the enhancing lesion was measured on post-contrast T1-weighted or subtracted axial images at peak enhancement. For both CESM and MRI, in the evaluations during NAC and post NAC, enhancing foci spread inside the tumor bed were considered as the expression of what remained of the same pathologic area, thus defined with a single overall measure. As this evaluation is already quite common in MRI, we considered it also for CESM, according to its high sensitivity and specificity described in the literature [7–16, 22–25].

According to the literature [22, 23] and the Breast Imaging-Reporting and Data System (BI-RADS) lexicon, the lesion location and a detailed descriptive analysis of its enhancement were reported both for MRI and CESM pre NAC and post NAC: *non*-*mass enhancement*, evaluating its distribution, or *mass enhancement*, defining lesion shape, margins and vascularization, considering both qualitative evaluation of contrast uptake and internal enhancement patterns.

### Surgical specimens

Tumor size was macroscopically measured in formalin-fixed specimens. Histopathological examination was performed in paraffin-embedded specimens. Tumor size was defined as the largest dimension based on macroscopic and histopathological examination. In the case of multifocal breast cancer, the maximum dimension of the largest invasive tumor was used.

In clinical practice the persistence of residual ductal carcinoma in situ (DCIS) components is considered as pathological complete response (pCR) [26]. However, in comparing CESM and MRI measurements with histopathological response on surgical specimens, we considered the DCIS component as part of the residual neoplasm, because when visible on imaging, it impacted surgical planning.

### Statistical analysis

In the absence of an a priori hypothesis, given the exploratory nature of the study, no formal sample size calculation was performed. Primary analysis included assessing agreement between CESM and MRI and histopathological assessment, and between the two diagnostic tools (pre, during and post NAC), measured with Lin’s coefficient [27]. The agreement difference estimate was accompanied by a bootstrap (bias-corrected and accelerated) confidence interval. In the first evaluation the Pearson’s correlation coefficient (PCC) was also calculated to allow easier comparison with similar previous studies in the literature.

Secondary analyses included estimation of the diagnostic performance indexes of each method for complete response with 95% confidence intervals (Clopper-Pearson-method [28]). Statistical analysis was carried out using R 3.2.3 (R Foundation for Statistical Computing, Vienna, Austria).

## Results

Of the initial 54 patients enrolled, 46 completed the study (8/54 were excluded because of interruption in NAC before any imaging monitoring could be performed). Table 1 shows detailed patient characteristics. No multifocal or multicentric tumors were detected.

**Table 1:** Characteristics of the patients that concluded the study

Characteristic	Value	
Number of included patients	46	
Age, mean (range), years	54 (33–72)	
Postmenopausal, *n* (%)	20	43
Histological assessment, *n* (%)		
Invasive ductal carcinoma	40	87
Invasive lobular carcinoma	4	9
Metaplastic carcinoma	2	4
TNM stage at diagnosis, *n* (%)		
T2N0	13	28
T2N+	17	37
T3N0	5	11
T3N+	9	20
T4N+	2	4
Molecular characteristics, *n* (%)		
Luminal A	3	6
Luminal B	16	35
Luminal B HER2+	6	13
HER2+	9	20
Triple negative	12	26
Histopathological response to NAC in surgical specimen (RECIST 1.1), *n* (%)		
Complete response (pCR)	8	17
Partial response (pPR)	32	70
Stable disease (pSD)	5	11
Progressive disease (pPD)	1	2
Surgical treatment, *n* (%)		
Lumpectomy	28	61
Mastectomy	18	39

*HER2* human epidermal growth factor receptor 2, *NAC* neoadjuvant chemotherapy, *RECIS*T response evaluation criteria in solid tumors, *pCR* pathological complete response, *pPR* pathological partial response, *pSD* pathological stable disease, *pPD* pathological progressive disease

Among the 46 patients considered, as decided in the Breast Unit Multidisciplinary Group, mainly because of poor tolerance to NAC, 5 women interrupted the chemotherapy 3 months after it was started, after the second step of the imaging evaluation, and directly underwent surgery. In these patients, the second CESM and MRI have been analyzed as “post NAC” and compared to the results of assessments of the surgical specimens. Of these, according to the RECIST 1.1 criteria, two patients achieved pCR, one pPR, one pSD and one pPD. No adverse reaction was observed either to iodinated contrast medium or to gadolinium contrast agent.

**Fig. 2:**
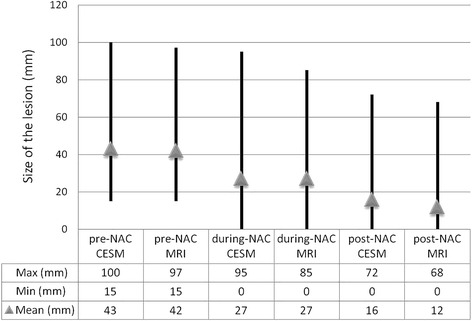
Mean tumor sizes measured on contrast-enhanced spectral mammography (CESM) and magnetic resonance imaging (MRI). NAC neoadjuvant chemotherapy

In a comparison of the post-NAC measurements with the histopathological response in surgical specimens, CESM had stronger agreement with histology than did MRI (Lin's coefficient 0.81 and 0.59, respectively; CESM-MRI concordance difference 0.22, CI 0.07–0.58; PCC 0.85 and 0.67, respectively). Mean tumor sizes measured on CESM and MRI are shown in Fig. 2. Both methods tended to underestimate the real extent of the residual tumor (mean underestimation of 4.1 mm in CESM and of 7.5 mm in MRI).

**Fig. 3:**
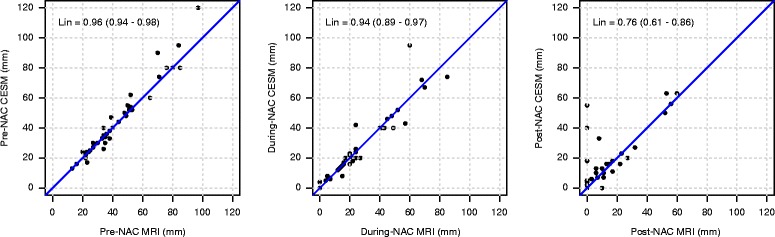
Agreement between measurements on contrast-enhanced spectral mammography (CESM) and magnetic resonance imaging (MRI) at each step of the study. Agreement (Lin’s coefficient) for measurement of tumor sizes using CESM and MRI (mm), respectively, before the beginning of (pre-NAC), during (during-NAC) and after the end of (post-NAC) neoadjuvant chemotherapy just before surgery. Progressive dimensional shrinkage and a difference between measurement sets using the two diagnostic tools can be observed

Measurements on CESM image sets agreed strongly with MRI, especially before (Lin’s coefficient 0.96, CI 0.94–0.98) and during (Lin’s coefficient 0.94, CI 0.89–0.97) NAC (Fig. 3). The post-NAC agreement coefficient was 0.76 (Lin’s coefficient, CI 0.61–0.86), revealing considerable divergence, which reflects the different correlation with histopathological assessment.

Post-NAC, 28% of patients had no residual pathological enhancement on CESM as compared to 46% on MRI.

According to histopathological assessment, 87% (40/46) of patients responded to NAC (pCR + pPR), with pCR achieved in 17% (8/46). CESM defined 40 patients as responders (CR + PR) while MRI defined 41. In this single discordance, the shrinkage of tumor was 25% on CESM and 32% on MRI, close to the threshold (30%). The main differences between CESM and MRI were observed in the distinction between CR and PR. Referring to histopathological response on surgical specimens, diagnostic performance indexes in pCR prediction are reported for both methods in Table 2.

**Table 2:** Diagnostic performance indexes in assessing complete response (CR) according to RECIST criteria 1.1, using CESM and MRI, compared to histopathological assessment of surgical specimens (gold standard)

Assessment				
	RECIST 1.1	Hystopathology pCR	Hystopathology non-pCR (pPR, pSD, pPD)	
CESM	CR	8	6	*PPV* 57% (CI 29–82%)
	non-CR (PR, SD, PD)	0	32	*NPV* 100% (CI 90–100%)
		*Sensitivity* 100% (CI 63–100%)	*Specificity* 84% (CI 69–94%)	
MRI	CR	7	15	*PPV* 32% (CI 14–55%)
	non-CR (PR, SD, PD)	1	23	*NPV* 96% (CI 79–100%)
		*Sensitivity* 87% (CI 47–100%)	*Specificity* 60% (CI 43–76%)	

*RECIST* response evaluation criteria in solid tumors, *CESM* contrast-enhanced spectral mammography, *MRI* magnetic resonance imaging, *pCR* pathological complete response, *pPR* pathological partial response, *pSD* pathological stable disease, *pPD* pathological progressive disease, *PR* partial response, *SD* stable disease, *PD* progressive disease, *PPV* positive predictive value, *NPV* negative predictive value

**Fig. 4:**
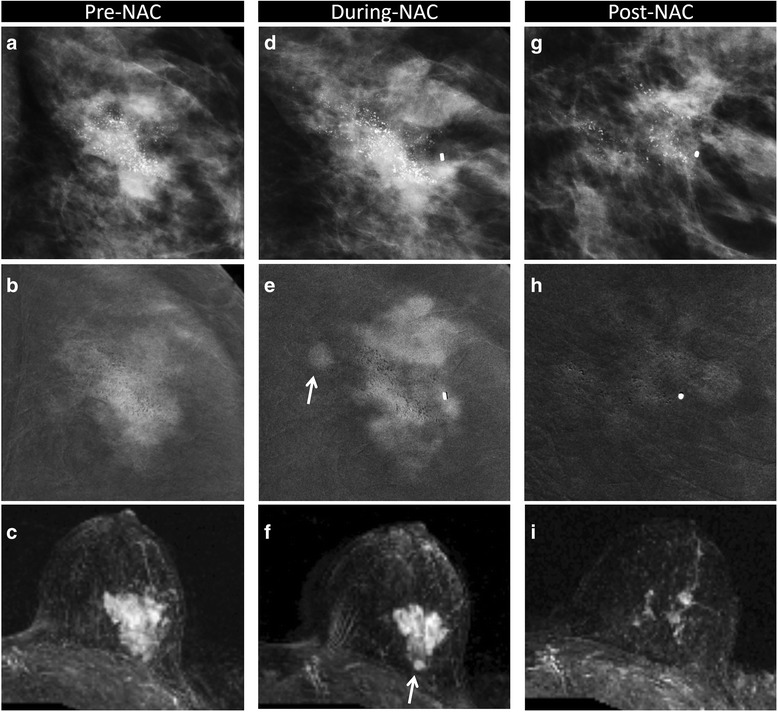
Partial response in invasive ductal carcinoma (IDC) and ductal carcinoma in situ (DCIS) component with microcalcifications. Partial response to neoadjuvant chemotherapy (NAC) in a 45-year-old woman with 50 mm in situ and IDC (G3, luminal B) in the left breast, correctly assessed using contrast-enhance spectral mammography (CESM) (**a**, **d**, **g** magnification of craniocaudal (CC) low-dose image; **b**, **e**, **h** magnification of CC recombined image) and magnetic resonance imaging (MRI) (**c**, **f**, **i** axial maximum intensity projection reconstruction). Pre-NAC, evaluation (**a**, **b**, **c**) showed the presence of microcalcifications (**a**) in the context of the enhancing tumor bed on CESM (**b**). MRI revealed 50 mm of malignant enhancement (**c**). During-NAC (**d**, **e**, **f**), both on CESM (**d**, **e**) and on MRI (**f**) we identified progressive disease with the comparison of a second satellite lesion (*arrow*). Post NAC (**g**, **h**, **i**), multiple foci of residual enhancement scattered inside the tumor bed were visible on CESM (**g**, **h**) and MRI (**i**). Partial response was confirmed by histopathological examination of the surgical specimen

On histopathological assessment 20 tumors had a DCIS component, mostly in the form of small foci in the tumor bed (Fig. 4). Of these, only three cases were classified as pCR since no concomitant residual invasive component was found: two were considered as CR both on CESM and MRI, whereas in just one case CESM revealed 5 mm of faint enhancement in the tumor bed, confirmed by histopathological assessment corresponding to a 3 mm DCIS. No surgical re-intervention was needed.

**Fig. 5:**
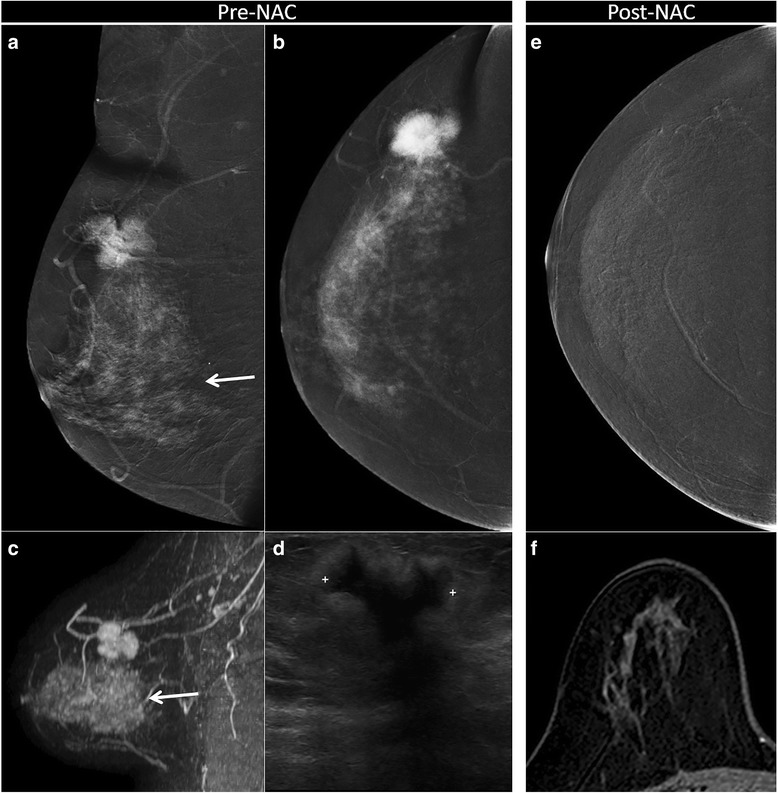
Complete response correctly assessed both with contrast-enhanced spectral mammography (CESM) and magnetic resonance imaging (MRI). Complete response to neoadjuvant chemotherapy (NAC) in a 44-year-old woman with 30 mm in situ and invasive ductal carcinoma (G3, human epidermal growth factor receptor 2 (HER2)^+^) in the right breast, correctly assessed with CESM (**a** medio-lateral oblique recombined image; **b**, **e** craniocaudal recombined image) and MRI (**c** sagittal maximum intensity projection reconstruction; **f** axial post-contrast T1-weighted at peak enhancement). Pre-NAC (**a**, **b**, **c**, **d**), evaluation showed the same polygonal shape, well-defined margins and same size on CESM (**a**, **b**), on MRI (**c**) and on ultrasound (**d**). Background parenchymal enhancement (arrow) was visible both on CESM (**a**) and MRI (**c**), with no significant influence on the assessment of the size of the lesion. Post-NAC, no residual pathological enhancement was visible on CESM (**e**) or on MRI (**f**). Complete response was confirmed by histopathological examination of the surgical specimen

**Fig. 6:**
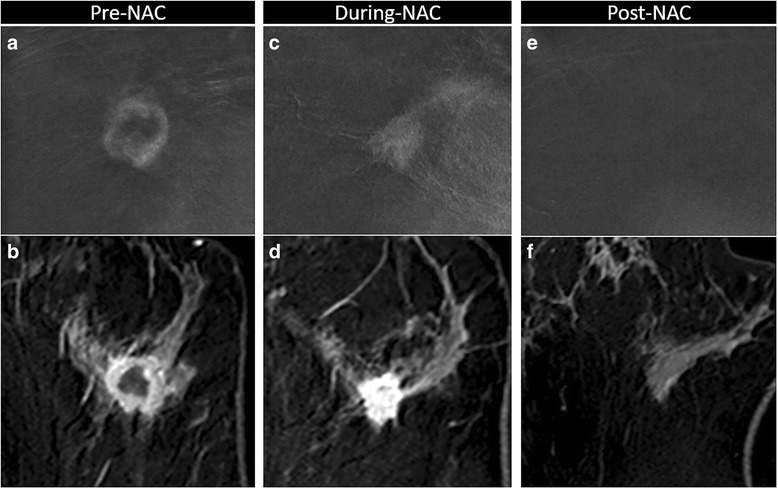
Variation of enhancement during neoadjuvant chemotherapy (NAC). Complete response to NAC in a 41-year-old woman with 27 mm invasive ductal carcinoma (G3, triple negative) in the left breast, correctly assessed with contrast-enhanced spectral mammography (CESM) (**a**, **b**, **c** magnification of craniocaudal recombined image) and magnetic resonance imaging (MRI) (**b**, **d**, **f** axial post-contrast T1-w eighted at peak enhancement). Pre-NAC, evaluation showed the same round shape, well-defined margins and rim enhancement on CESM (**a**) and on MRI (**b**). During-NAC, both on CESM (**c**) and on MRI (**d**) we noted concentric shrinkage, and the tumor was no longer rimmed, with no significant loss of enhancement intensity. Post-NAC, no residual pathological enhancement was visible on CESM (**e**) or on MRI (**f**). Complete response was confirmed by the histopathological examination of the surgical specimen

**Fig. 7:**
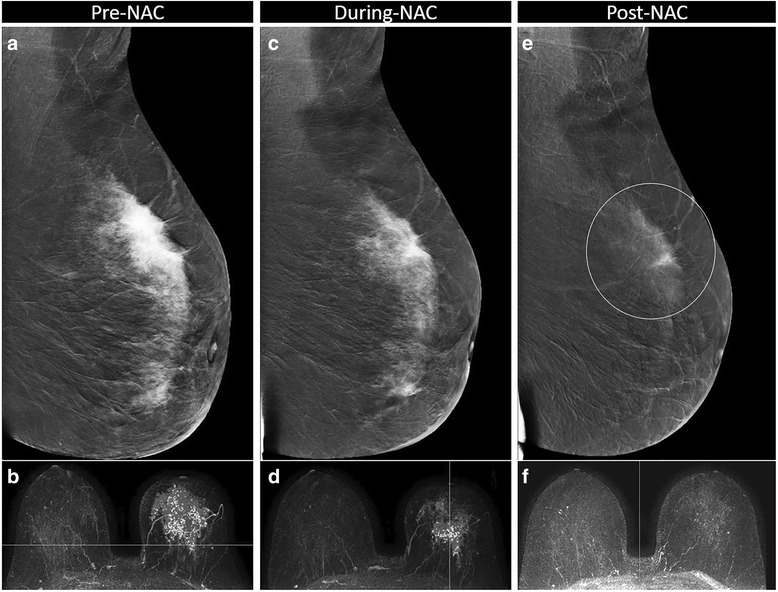
Partial response correctly assessed with contrast-enhanced spectral mammography (CESM) but misinterpreted as a complete responder with MRI. Partial response to neoadjuvant chemotherapy (NAC) in a 48-year-old woman with a 97-mm invasive lobular carcinoma, luminal B, in the left breast, misinterpreted as a complete responder by MRI. Pre-NAC, evaluation showed wide non-focal enhancement on CESM (**a** medio-lateral oblique (MLO) recombined image) and on MRI (**b** axial maximum intensity projection (MIP) reconstruction). During-NAC, a concentric shrinkage and loss of enhancement intensity was visible on CESM (**c** MLO recombined image) and on MRI (**d** axial MIP reconstruction). Post-NAC, no pathological enhancement was visible on MRI (**f** axial MIP reconstruction), whereas in CESM (**e** MLO recombined image) faint enhancement in a part of the tumor bed (circle) was still visible, considered as residual tumor. The patient underwent mastectomy, and a partial response was confirmed by the histopathological examination of the surgical specimen, with 55 mm of residual invasive lobular carcinoma

Table 3 describes the tumor characteristics at diagnosis and after NAC, according to the BI-RADS lexicon. During and after chemotherapy, we mainly observed progressive tumor shrinkage and loss of enhancement among the responders (Fig. 5). Focusing on CESM, the shrinkage of the lesion was concentric in 60% of patients (Fig. 6), while in 40% there were multiple small foci of faint enhancement included in the tumor bed (Fig. 7).

**Table 3:** Tumor characteristics at diagnosis and variations after NAC

	Pre-NAC CESM	Pre-NAC MRI	Post-NAC CESM	Post-NAC MRI
Mass enhancement	89% (41/46)	89% (41/46)		
*Shape*				
Round	24% (11/46)	26% (12/46)	15% (7/46)	15% (7/46)
Oval	26% (12/46)	26% (12/46)	22% (10/46)	11% (5/46)
Irregular	39% (18/46)	37% (17/46)	26% (12/46)	22% (10/46)
None			26% (12/46)	41% (19/46)
*Margins*				
Circumscribed	46% (21/46)	48% (22/46)	35% (16/46)	24% (11/46)
Non-circumscribed	43% (20/46)	41% (19/46)	28% (13/46)	24% (11/46)
None			26% (12/46)	41% (19/46)
Non-mass enhancement	11% (5/46)	11% (5/46)		
*Distribution*				
Regional	9% (4/46)	9% (4/46)	7% (3/46)	4% (2/46)
Segmental	2% (1/46)	2% (1/46)	2% (1/46)	2% (1/46)
None			2% (1/46)	4% (2/46)
Enhancement pattern				
Heterogeneous	83% (38/46)	80% (37/46)	61% (28/46)	43% (20/46)
Clustered ring	17% (8/46)	20% (9/46)	11% (5/46)	11% (5/46)
None			28% (13/46)	46% (21/46)
Contrast uptake				
Strong	78% (36/46)	83% (38/46)	2% (1/46)	4% (2/46)
Intermediate	22% (10/46)	17% (8/46)	63% (29/46)	48% (22/46)
Weak			7% (3/46)	2% (1/46)
None			28% (13/46)	46% (21/46)

*NAC* neoadjuvant chemotherapy, *CESM* contrast-enhanced spectral mammography, *MRI* magnetic resonance imaging

As none of the study patients had evidence of bilateral neoplasms at the pre-NAC evaluation, the CESM examinations were always mono-lateral. The total mean AGD of a complete mono-lateral CESM, consisting of low-energy and high-energy exposures on two mammographic views (CC and MLO), was 7.15 mGy (range 3.88–23.34 mGy), depending on breast thickness (mean of 59 mm, range 40–85 mm) and glandularity, considered as the percentage of peak breast density measured automatically by the mammographic unit (mean of 50%, range 9–95%).

## Discussion

In this prospective study we hypothesized that CESM could provide an accurate assessment of tumor response after NAC, comparable to that provided by MRI, which is currently considered the most reliable diagnostic method in treatment monitoring. In this study, the correlation between measurements using these imaging tools and histopathological assessment, the gold standard, was stronger for CESM (Lin’s coefficient 0.81; PCC 0.85) than for MRI (Lin’s coefficient 0.59; PCC 0.67), and both imaging tools involved slight underestimation.

In women who did not undergo NAC, Lobbes et al. [13] identified PCC > 0.9 with both CESM and MRI compared to histopathological assessment, with a small systematic overestimation of the tumor diameter measured on MRI, but not on CESM. In a similar setting, Łuczyńska et al. [11] described overestimation of lesion size both in CESM (1.7 mm) and in MRI (1.8 mm). Fallenberg et al. [16] identified underestimation of the tumor size using MRI compared to CESM and pathological assessment, describing an increasing discrepancy in the size of the pathological lesion in larger breast tumors measured by the imaging methods. This evaluation may also be relevant to the patients enrolled in our study, as NAC is recommended for locally advanced breast carcinomas, and at diagnosis the mean size of the lesions was 43 mm.

Several variables influence tumor shrinkage and its evaluation during NAC, including primary tumor size, edema or necrosis. Also, the various chemotherapeutic drugs contribute differently to the variation in the primitive lesion, with significant consequences on imaging. Taxanes have been reported to have greater antiangiogenic activity compared to anthracyclines [29], and this antivascular effect, with the lack of surrounding inflammatory response, lowers the enhancement on tumor tissue, leading to underestimation of tumor size by MRI [5]. These effects were similar in our patients as the NAC protocol was the same, including both anthracyclines and taxanes.

In this study, the cumulative impact of these variables is reflected in the progressively weaker correlation between measurements using CESM and MRI, comparing the pre-NAC (Lin’s coefficient 0.96) and during-NAC (Lin’s coefficient 0.94) evaluations to the final post-NAC (Lin’s coefficient 0.76). Apart from the changes in tumor micro-vessel functionality, it must be considered that the overall loss of cellularity is not always reflected by a decrease in tumor size, because the fibrous stroma remains [30]. Furthermore, when the residual cancer cells appear as small foci or scattered cells, they receive nutrients via diffusion and not from vascular perfusion [30]. Similarly, the contrast agent also moves to the ducts by diffusion, so the amount of contrast reaching the tissue is time dependent. Fallenberg et al. [16] observed that longer time delays between contrast injection and CESM exposure could result in stronger enhancement and hence better visibility compared to MRI. In contrast to the rapid washout in MRI, enhancement on CESM in fact persists for at least 10 min after contrast agent infusion [15]. Further studies are encouraged to improve a protocol for CESM dedicated to NAC monitoring, especially focusing on the optimal timing in CESM acquisition after NAC to stress the via-diffusion enhancement. Other possible variables include differences in the molecular structure of gadolinium-based and iodine-based contrast medium and the effects of breast compression. And last of all, the 10 times higher spatial resolution of CESM versus MRI [11] may help reveal tiny foci of residual enhanced lesion on the tumor bed.

According to the RECIST 1.1 criteria, the main issue considered was the distinction between CR and PR, with a significant divergent post-NAC assessment of no residual pathological enhancement on CESM (30%) and on MRI (48%). In the limited population of this study, CESM showed promising results in the definition of CR, correctly assessing each pCR, whereas one was missed on MRI. Otherwise, each patient with residual disease enhanced in CESM was confirmed on assessment of the surgical specimen.

The reliable assessment of both the extent of residual disease and pCR after NAC is crucial to decision making, surgical planning and prediction of final outcomes. Any overestimation of lesion sizes would have less severe consequences on surgical treatment than would underestimation, especially regarding safety margins. However the underestimation of residual tumor in this study had no consequences on the surgical planning since it mainly affected the discrimination between PR and CR in responding patients, in whom the tumor bed had to be completely excised anyway.

The advantages of CESM over breast MRI are the short examination time and lower costs [14]. Furthermore, CESM permits evaluation of both microcalcifications (on the low-energy image) and of enhanced structure (on the recombined image) in a single examination, overcoming this limitation of MRI and its variable sensitivity to in situ cancers [11, 31]. Even if the persistence of residual DCIS is considered as pCR, we believe that its extent must be considered for correct surgical planning, and CESM may be a precious tool for this purpose. Further evaluations are needed. CESM might also be a good alternative if MRI is not available or for patients with contraindications to MRI (e.g. pacemakers) or claustrophobia. The tolerance and overall preference of CESM versus MRI [13] must be taken into serious consideration in women who are facing the psychological burden of chemotherapy and have to repeat the examination three times in approximately 6 months. Disadvantages are the use of an iodine-based contrast agent instead of a gadolinium-based agent and the additional radiation dose. In this study the mean AGD of a complete mono-lateral CESM, consisting of low-energy and high-energy exposures for two mammographic views (CC and MLO), was 7.15 mGy. These doses are close to the ranges described in the literature [9, 16, 32, 33]. The AGD for the low-energy image was equivalent to that of one conventional mammogram, while for the high-energy image it was approximately 20% of the dose of one conventional mammogram [9]. However, this added dose was not considered as restricting, since all patients enrolled also had indications for radiotherapy treatment, which includes a total dose of 45 Gy on the target, i.e. about 10,000 times the dose of a CESM. In this study our ethical preference was to limit CESM to the affected breast to avoid any additional dose.

This study had several limitations. First of all, the small number of patients enrolled who were not randomized, which limits the statistical significance of the results. Nevertheless, the promising findings encourage further studies. Second, MRI was performed using different units, which may have led to heterogeneous findings despite following the same protocol. Also, the participation of seven independent radiologists might be considered a limitation because of a supposed non-homogeneous interpretation of images. However, this reflects the routine diagnostic workflow. Finally, no multicentric or multifocal breast cancers were included in the study.

## Conclusions

CESM and MRI lesion size measurements were highly correlated. CESM seems at least as reliable as MRI in assessing the response to NAC, with encouraging results, and may be considered as an alternative if MRI is contraindicated or its availability is limited.

## Abbreviations

AGD: Average glandular dose
BIRADS: Breast Imaging Reporting and Data System
CAD: Computer-aided detection
CC: Craniocaudal
CESM: Contrast-enhanced spectral mammography
CI: Confidence interval
CR: Complete response (RECIST 1.1 criteria)
DCIS: Ductal carcinoma in situ
IDC: Invasive ductal carcinoma
HER2: Human epidermal growth factor receptor 2
MIP: Maximum intensity projection
MLO: Medio-lateral oblique
MRI: Magnetic resonance imaging
NAC: Neoadjuvant chemotherapy
PCC: Pearson correlation coefficient
pCR: Pathological complete response (RECIST 1.1 criteria)
PD: Progressive disease (RECIST 1.1 criteria)
pPD: Pathological progressive disease (RECIST 1.1 criteria)
pPR: Pathological partial response (RECIST 1.1 criteria)
PR: Partial response (RECIST 1.1 criteria)
pSD: Pathological stable disease (RECIST 1.1 criteria)
RECIST: Response evaluation criteria in solid tumors
SD: Stable disease (RECIST 1.1 criteria)
US: Ultrasound
